# Interactions of Intestinal Bacteria with Components of the Intestinal Mucus

**DOI:** 10.3389/fcimb.2017.00387

**Published:** 2017-09-05

**Authors:** Jean-Félix Sicard, Guillaume Le Bihan, Philippe Vogeleer, Mario Jacques, Josée Harel

**Affiliations:** ^1^Centre de Recherche en Infectiologie Porcine et Aviaire, Faculté de Médecine Vétérinaire, Université de Montréal Saint-Hyacinthe, QC, Canada; ^2^Regroupement de Recherche Pour un Lait de Qualité Optimale (Op+Lait), Faculté de Médecine Vétérinaire, Université de Montréal Saint-Hyacinthe, QC, Canada

**Keywords:** mucus, commensals, pathogens, biofilm, microbiota, microflora, goblet cells

## Abstract

The human gut is colonized by a variety of large amounts of microbes that are collectively called intestinal microbiota. Most of these microbial residents will grow within the mucus layer that overlies the gut epithelium and will act as the first line of defense against both commensal and invading microbes. This mucus is essentially formed by mucins, a family of highly glycosylated protein that are secreted by specialize cells in the gut. In this Review, we examine how commensal members of the microbiota and pathogenic bacteria use mucus to their advantage to promote their growth, develop biofilms and colonize the intestine. We also discuss how mucus-derived components act as nutrient and chemical cues for adaptation and pathogenesis of bacteria and how bacteria can influence the composition of the mucus layer.

## Introduction

**Figure 1:**
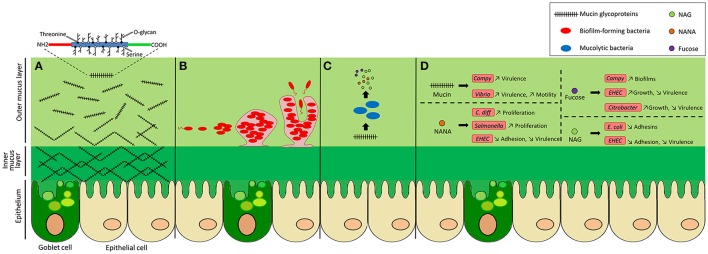
Bacterial activities in the colonic mucus layer environment. The colonic epithelium is covered by a mucus gel layer formed of glycoproteins called mucins. **(A)** Mucins consist of a protein core and a high number of O-linked glycans. They are secreted by goblet cells and are assembled into a net-like structure that forms a dense inner layer, firmly attached to cells, that does not allow bacteria to penetrate. Further from the epithelium, the outer layer becomes loose and permissive, providing a niche for intestinal bacteria. **(B)** Mucus oligosaccharides can act as adhesion sites for bacteria, facilitating their colonization. Some bacteria are able to form microcolonies and biofilms. **(C)** Bacteria with mucolytic activity can release monosaccharides from mucin O-glycans and metabolize them. These sugars can also be utilized by nearby bacteria. **(D)** Mucus components can influence the behavior of pathogenic bacteria by increasing or decreasing their virulence expression, adhesion, motility, proliferation, or growth.

The gastrointestinal tract harbors a complex bacterial community called the intestinal microbiota that, in healthy conditions, maintains a commensal relationship with our body. Various mechanisms are used by the host to keep intestinal homeostasis and to prevent aberrant immune responses directed against the microbiota. One of these is the production of a mucus layer that covers the epithelial cells of the gut. This mucus is synthesized and secreted by host goblet cells and form an integral structural component of the mammal intestine. Its major function is to protect the intestinal epithelium from damage caused by food and digestive secretions (Deplancke and Gaskins, 2001). The mucus layer provides a niche for bacterial colonization because it contains attachment sites and is also a carbon source (Harel et al., 1993). Effectively, the mucus is a direct source of carbohydrates that are released in the lumen. Therefore, several bacterial species of the microbiota can use mucus glycan as a carbon source (Ouwerkerk et al., 2013). An alteration in glycan availability modifies the composition of the microbiota (Martens et al., 2008). The mucus layer also prevents pathogens from reaching and persisting on the intestinal epithelial surfaces and thereby is a major component of innate immunity. It is constantly renewed and acts as a trap for commensal residents, but also for pathogens, preventing their access to the epithelia (Johansson et al., 2008; Bertin et al., 2013). Although its composition and thickness vary along the gut, the mucus layer is mainly formed of glycoproteins containing different glycans; nonspecific antimicrobial molecules, such as antimicrobial peptides (AMP); secreted antibodies targeting specific microbial antigens; and other intestinal proteins (McGuckin et al., 2011; Antoni et al., 2014). Interaction with the mucus layer is important for the colonization of gut commensals as well as some pathogens that have evolved to adhere to mucus and exploit it (Juge, 2012). Some pathogens also use mucus components as a cue to modulate the expression of virulence genes and thereby adapt to the host environment. In this Review, we describe the interactions between bacteria and components of the human mucus layer: their use as carbon sources, adhesion sites and their genetic adaptation (Figure 1).

## The gastrointestinal mucus

### Mucus composition

The intestinal mucus is composed mainly of mucins that are complex agglomerates of structural glycoproteins with specific O-linked glycans (O-glycans) produced by specialized cells of the host called goblet cells (Forstner, 1995). Mucins can either be secreted and form a gel, or be produced as membrane-bound glycoproteins that are part of the epithelial glycocalyx (Johansson et al., 2008, 2011; Jonckheere et al., 2013; Nilsson et al., 2014). These glycoproteins share a common structure made of tandem repeated amino acids rich in proline, threonine and serine and are call PTS domains. These sequences of amino acid provide sites for the covalent attachment of the polysaccharides and are widely *O*-glycosylated (Moran et al., 2011). Four different types of polysaccharide core structures are commonly found in mucin glycoproteins. These cores are formed by a combination of three polysaccharides, galactose, N-acetyl-galactosamine and N-acetyl-glucosamine (Larsson et al., 2009; Juge, 2012). Different chains of glycan will be attached to the core. The terminal monosaccharide is usually a fucose or a sialic acid (Larsson et al., 2009; Juge, 2012). Oligosaccharide chains are also sulfated, especially in colonic regions (Rho et al., 2005). The mucin proteins MUC1, MUC5AC, and MUC6 mainly form the mucus layer in the stomach, whereas MUC2 is the most abundant mucin in the small intestine and the colon (Johansson et al., 2009; Moran et al., 2011). The thickness of the mucus layer varies through the gut. The colon, which harbors the highest density of microorganisms, is covered by the thickest mucus layer (Gum et al., 1994). It is composed of an inner layer that is dense and firmly attached to the epithelium and an outer loose layer that is exposed to bacterial proteolytic activity. The numerous O-glycans of the outer layer can serve as adhesion sites and as nutrients for bacteria while the inner layer is less permissive to bacterial penetration in healthy individuals (Johansson et al., 2008, 2011). Most bacterial residents are present in the outer mucus layer and the competition for survival in this niche shapes the composition of the microbiota. The differential resource utilization of bacterial species participates to the establishment of distinct communities that includes non-mucolytic bacteria (Li et al., 2015).

### Role of the mucus layer

The mucus barrier has an important role in regulating the severity of infectious diseases. It provides protection against many intestinal pathogens, including *Yersinia enterocolitica, Shigella flexneri, Salmonella*, and *Citrobacter rodentium* (Mantle and Rombough, 1993; Bergstrom et al., 2010; Arike and Hansson, 2016). MUC2 (Mouse, Muc2) plays a crucial role during infection. Using *Muc2*-deficient mice, it was shown that the glycoprotein is critical in controlling *Salmonella* infection (Zarepour et al., 2013). Moreover, *Muc2*^−/−^ mice revealed higher susceptibility to attaching and effacing (A/E) *Citrobacter rodentium* infections (Bergstrom et al., 2010).

An alteration of mucosal integrity is generally associated with health problems, such as inflammatory bowel diseases, including ulcerative colitis and Crohn's disease (Trabucchi et al., 1986; Hanski et al., 1999). During ulcerative colitis, alteration of mucus integrity results in a thinner mucus layer due to goblet cell depletion (Pullan et al., 1994) and a reduced O-glycosylation and sulfation of mucins (Raouf et al., 1992; Larsson et al., 2011). During Crohn's disease, the mucus layer is essentially continuous and comparable to healthy mucosa (Strugala et al., 2008) although there is evidence of abnormal expression and glycosylation of the mucin (Buisine et al., 2001; Moehle et al., 2006; Dorofeyev et al., 2013). These changes in the mucosal environment could also be linked to dysbiosis, an abnormal change in the composition of the intestinal microbiota due to Crohn's disease. Once impaired, the mucus barrier becomes permeable to bacteria that are able to access the epithelium and therefore cause inflammation (Antoni et al., 2014; Johansson et al., 2014), which is why the integrity of the mucus layer is critical for the upkeep of a homeostatic relationship between the intestinal microbiota and its host.

## Mucin as a growth substrate

Mucin proteins are highly glycosylated and therefore constitute a carbon and energy source for intestinal microbiota. A key nutritional aspect of the mucus layer for gut bacteria is its high polysaccharide content with up to 80% of the mucin biomass being composed of mostly O-linked glycans (Johansson et al., 2009, 2011; Marcobal et al., 2013).

### Mucolytic bacteria

A distinct subset of intestinal bacteria possesses the enzymatic activity, such as glycosidases, necessary for the degradation of mucin oligosaccharides, which can be further metabolized by resident microbiota (Koropatkin et al., 2012; Ouwerkerk et al., 2013). Indeed, various anaerobic bacteria species of gut microbiota, such as *Akkermansia muciniphila* (Derrien et al., 2004; Png et al., 2010), *Bacteroides thetaiotaomicron* (Xu et al., 2003; Sonnenburg et al., 2005), *Bifidobacterium bifidum* (Crociani et al., 1994; Png et al., 2010; Garrido et al., 2011), *Bacteroides fragilis* (Macfarlane and Gibson, 1991; Swidsinski et al., 2005a; Huang et al., 2011), *Ruminococcus gnavus* (Png et al., 2010; Crost et al., 2013), and *Ruminococcus torques* (Hoskins et al., 1985; Png et al., 2010) are now known as mucin-degrading specialists. These bacteria will use their specific enzymatic activities to release monosaccharides attached to the mucin glycoproteins. Some mucolytic bacteria, such as *B. thetaiotaomicron*, that possess an important variety of glycosidases, are better suited for the utilization of a wide range of glycans (Xu et al., 2003; Marcobal et al., 2013). To complete the degradation of mucins, a combination of enzymatic activity of several mucolytic bacteria is needed (Derrien et al., 2010; Marcobal et al., 2013). Therefore, MUC2 glycans act as nutritional sources for bacteria that can utilize the mucus-derived sugars, but lack the enzymes necessary for cleaving sugar linkages (Johansson et al., 2015; Arike and Hansson, 2016). Commonly, several bacteria collaborate in a community and it has been shown that the sulfatase activity of some commensal bacteria on sulfomucin allows glycosidases to access and act on mucins (Rho et al., 2005). Released saccharides, such as *N*-acetyl-D-glucosamine (GlcNAc also called NAG), *N*-acetylgalactosamine (GalNAc), galactose, fucose and sialic acid (*N*-acetylneuraminic acid also called NANA) can then be used by the degrader itself or by other resident bacteria (Bjursell et al., 2006; Martens et al., 2008; Sonnenburg et al., 2010). As example, commensal *E. coli* that are limited to growth on mono- or disaccharides, are unable to degrade the complex polysaccharides that constitute mucin (Hoskins et al., 1985) and therefore use such carbohydrate sources (Chang et al., 2004; Png et al., 2010; Bertin et al., 2013). Another example is vancomycin-resistant *Enterococcus* that can grow on mucin pre-digested with extracts from human stools, but not on purified mucin. This suggests that *Enterococcus* can benefit of the microbiota activity on mucin and uses released mucus-derived products (Pultz et al., 2006). In this way, mucolytic bacteria make mucus O-glycan derived products also available for other bacterial residents.

### Use of mucus-derived nutrients by pathogens

Intestinal pathogens have developed strategies to compete with commensal microflora for nutrients, such as carbohydrates and these strategies have been reviewed in Conway and Cohen (2015), Vogt et al. (2015), and Baumler and Sperandio (2016). Pathogenic and commensal *E. coli* strains displayed considerable catabolic diversity when colonizing streptomycin-treated mice, indicating that nutrient availability can influence their colonization success and their niche adaptation (Maltby et al., 2013). For example, pathogenic *E. coli* such as enterohemorrhagic *E. coli* (EHEC) strain EDL933 efficiently utilizes some mucus-derived monosaccharides. This can provide competitive growth compared to that of commensal *E. coli* (Fabich et al., 2008). Moreover, the metabolic flexibility of some pathogenic strains to use both glycolytic and gluconeogenic nutrients may be advantageous (Bertin et al., 2013). The pathogen *Vibrio cholerae*'s preferential use of mucus-derived monosaccharides, such as GlcNAc and sialic acid confers an advantage in the infant mouse model of infection (Almagro-Moreno et al., 2015). *C. jejuni* also possess the ability to metabolize fucose. Its growth is enhanced in culture medium supplemented with it (Alemka et al., 2012). In addition, antibiotic treatment also perturbs the microbiota and therefore affects the availability of mucin carbohydrates. The concentration of free fucose and sialic acid reaching high levels during antibiotic treatment facilitates expansion of pathogens such as *Salmonella enterica* serotype Typhimurium and *Clostridium difficile* (Ng et al., 2013). In addition, *Salmonella* serotype Typhimurium is known both to bind glycoprotein containing sialic acids (Vimal et al., 2000) and to have the ability to release the carbohydrate using its sialidase (Hoyer et al., 1992). Thereby, to colonize specific niches, many pathogens have evolved in a way to use mucus-derived sugars as a carbon source.

## Bacterial adhesion to mucins

Mucins proteins are highly glycosylated. Their O-glycans are used as ligands for bacterial adhesins (Juge, 2012). It can be speculated that adhesion to mucins may initiate colonization of the intestine. The carbohydrate structures on mucins can provide initial attachment site to bacteria including specialized pathogens and could facilitate the invasion of epithelial cells (Derrien et al., 2010). As example, pathogenic microorganisms, such as *Campylobacter* and enterotoxinogenic *E. coli* (ETEC) are known to adhere to the glycoprotein MUC1 that is present in human breast milk. This interferes with colonization of these pathogens in the infant GI tract (Martin-Sosa et al., 2002; Ruiz-Palacios et al., 2003). Although no specific mucus-adherent microflora was identified (van der Waaij et al., 2005), there are evidence that bacteria can bind directly to mucins by expressing specific proteins, pili, fimbriae and flagella (Table 1).

**Table 1:** Bacterial adhesion to mucin components.

**Bacteria**		**Adhesin**	**Mucin glycoprotein**	**Mucin component**	**References**
**COMMENSAL BACTERIA**					
*Bacteroides fragilis*					Huang et al., 2011
*Bifidobacterium bifidum*		Extracellular transaldolase			Marcobal et al., 2013
*Bifidobacterium longum* subsp. *infantis*		Family 1 of solute binding proteins		Mucin oligosaccharides	Garrido et al., 2011
*Escherichia coli* Nissle 1917		Flagellum			Troge et al., 2012
Lactic acid bacteria		MUB			Boekhorst et al., 2006
		Pili			Kankainen et al., 2009; Le et al., 2013
**PATHOGENS**					
*Campylobacter jejuni*		Carbohydrate-lectin, FlaA, MOMP	MUC2		Tu et al., 2008; Naughton et al., 2013; Mahdavi et al., 2014
*Clostridium difficile*		FliC		Cecal mucus	Tasteyre et al., 2001
		FliD			
*Escherichia coli*	UPEC CFT073	F9 fimbriae		Galβ1-3GlcNAc structures	Wurpel et al., 2014
	EPEC E2348/69	H6 flagella	MUC2	Mucin-type core 2 O-glycan	Erdem et al., 2007; Ye et al., 2015
	EHEC EDL933	H7 flagella	MUC2	Mucin-type core 2 O-glycan	Erdem et al., 2007; Ye et al., 2015
*Listeria monocytogenes*		LPXTG-internalin proteins (MucBP) LmiA			Bierne et al., 2007; Mariscotti et al., 2014
*Salmonellae enterica* serotype Typhimurium		Fimbrial adhesin (std operon)		Alpha1-2 fucosylated receptor(s)	Chessa et al., 2009
*Vibrio cholerae*		Vibrio polysaccharide (VPS)			Liu et al., 2015
		Chitin-binding protein (GbpA)		N-acetyl D-glucosamine	Bhowmick et al., 2008

### Interactions between mucin and surface proteins

To adhere to mucus, commensal and pathogenic bacteria use different strategies. First, they can produce proteins that specifically bind the mucus. Mucus-binding proteins (MUB) are cell-surface proteins mainly described in lactic acid bacteria (LAB) (Boekhorst et al., 2006), especially in *Lactobacillus reuteri* (Roos and Jonsson, 2002; MacKenzie et al., 2009). MUB contain domains that are similar to the model mucin-binding protein (MucBP) from the Pfam database (Boekhorst et al., 2006). The MucBP domain is found in a variety of bacterial proteins that are known for their capacity to adhere to mucus (Juge, 2012). MUB also share structural and functional homology with pathogenic Gram-positive adhesins that have specificity to sialylated mucin glycans (Etzold et al., 2014). For example, some surface proteins of *Listeria monocytogenes* contain a MucBP domain similar to those found in *Lactobacillus*, allowing them to adhere to mucin (Bierne et al., 2007; Mariscotti et al., 2014). The causative agent of cholera, *V. cholerae*, can also bind to mucin using surface protein called GbpA (chitin-binding protein) that binds specifically to N-acetyl D-glucosamine residues of intestinal mucins (Bhowmick et al., 2008). In addition, *C. jejuni* is well-known for its ability to interact with different human histoblood group antigens (HBGAs) expressed in mucosa (Naughton et al., 2013). The major outer membrane protein (MOMP) of *C. jejuni* is involved in these interactions (Mahdavi et al., 2014). This way, *C. jejuni* can interact with intestinal mucin MUC2 in the intestine (Tu et al., 2008). Furthermore, *Bifidobacterium* spp. is also known for its specific adhesion to mucus. For example, in a *B. bifidum* mucin-binding assay, the expression of an extracellular transaldolase correlated with a positive mucin-binding phenotype (Gonzalez-Rodriguez et al., 2012). *B. longum* subsp. *infantis* is another species that binds specifically to mucin using family-1 solute binding proteins (Kankainen et al., 2009). Interestingly, a study using gnotobiotic mice colonized by *B. fragilis* and *E. coli* revealed that the commensal bacterium *B. fragilis* was found in the mucus layer while *E. coli* was only found in the lumen. Further analysis showed that *B. fragilis* specifically binds to highly purified mucins. This indicated that a direct bond with intestinal mucus could be a mechanism used by *B. fragilis* for gut colonization (Huang et al., 2011).

### Interactions between mucin and pili/fimbriae

In addition to produce specific mucus binding proteins, some bacteria can also use cell-surface appendix, such as pili or fimbriae to bind the mucus. For example, production of pili by LAB was shown to be implicated in mucus-binding activity (Douillard et al., 2013) and moreover, the SpaC pilus protein of *L. rhamnosus* GG was shown to strongly binds the human mucins (Kankainen et al., 2009). An *in vitro* study using mucus-secreting HT29-MTX intestinal epithelial cell model showed that the adhesion of *Salmonellae enterica* serotype Typhimurium to mucus-secreting intestinal epithelial cells was higher than in non- and low-mucus producing cells (Gagnon et al., 2013). Moreover, virulent strains seem to bind more efficiently to mucus than avirulent strains and the binding that preferentially targets the neutral mucin is mannose-dependant (Vimal et al., 2000). As with some uropathogenic *E. coli* (Wurpel et al., 2014), the adhesion of *S. enterica* serotype Typhimurium could be the result of interaction between fimbrial adhesin and mucin glycans, more specifically terminal fucose residues (Chessa et al., 2009). The *E. coli* K88 (F4) fimbriae is also able to bind mucus from the small intestines of 35-day-old piglets with a specificity to the glycolipid galactosylceramide (Blomberg et al., 1993). Hence, pili and fimbriae are involved in specific adhesion to mucus.

### Interactions between mucin and flagella

Many enteric bacteria also produce flagellum. In addition to their role in motility, flagella are also involved in adhesion. As example, the *E. coli* probiotic strain Nissle 1917 was shown to be able to interact, via its flagella, with human and porcine mucus but not with murine mucus. Furthermore, the mucus component gluconate has been identified as one receptor for the adhesion of these flagella (Troge et al., 2012). Other studies have revealed the role of the flagella for the binding of mucin glycoproteins by *C. difficile* (Tasteyre et al., 2001) and pathogenic *E. coli* (Erdem et al., 2007). Indeed, a mutation of the flagellum element *fliC* prevents the adhesion of EPEC and EHEC to mucins (Erdem et al., 2007). More recently, the flagella of EPEC (O127:H6) and EHEC (O157:H7) were shown to adhere to mucin-type core 2 O-glycan in MUC2. *C. jejuni* is another pathogen that uses its flagella to bind mucin. It was showed that the major flagella subunit protein (FlaA) is also involved in the adhesion to HBGA in the mucus. Therefore, flagella can be used in attachment strategies by gut residents.

## Bacterial biofilm and mucus

There are more mucus-associated bacteria in the proximal region of the colon than in distal colonic sites. Among the complex microbial communities within the gut, some are believed to form mucosal biofilm, that is a complex and self-produced polymeric matrix where microorganisms can attach to each other and be attached to the mucosal surface (de Vos, 2015). The rapid growth of the intestinal mucus and the lack of effective preservation techniques complicated the study investigating biofilms in healthy individuals (Bollinger et al., 2007; de Vos, 2015). However, biofilms were observed in artificial mucin gels that simulate the proximal and distal colon (Macfarlane et al., 2005), and also by electron microscopy in uninflamed proximal large bowel of mice (Swidsinski et al., 2005a), rat, baboon, and humans (Palestrant et al., 2004). Some evidence, such as the rates of plasmids transfer and the expression of colonization factors by gut bacteria, plead for the presence of biofilms in the gut (Macfarlane et al., 1997; Licht et al., 1999; Hooper and Gordon, 2001). In addition, components of the mucus layer, such as secretory IgA (SIgA) and mucins are likely to play a role in biofilm formation as they have been shown to modulate biofilm production *in vitro* (Bollinger et al., 2003, 2006; Slizova et al., 2015). Moreover, adherence of bacteria to mucin proteins could lead to growth of microcolonies that could further develop into biofilms (Kleessen and Blaut, 2007). Biofilms could also be formed on the surface of intestinal or gastric epithelia and interact with the secreted or membrane-bound mucins.

Alteration of the mucus layer occurs in cases of inflammatory bowel diseases (Bodger et al., 2006; Baumgart et al., 2007; Sheng et al., 2012). The increased presence of *B. fragilis* group and *Enterobacteriaceae* and their ability to form biofilms could play a role in these diseases (Swidsinski et al., 2005b, 2009). Within the *Enterobacteriaceae* family, the adherent-invasive *E. coli* (AIEC) strains associated with Crohn's disease (Masseret et al., 2001; Darfeuille-Michaud et al., 2004; Eaves-Pyles et al., 2008; Martinez-Medina et al., 2009a), are shown to be higher biofilm producers than non-AIEC strains (Martinez-Medina et al., 2009b). As with inflammatory bowel diseases, impaired mucin production is related to colorectal cancer (Weiss et al., 1996; Kim and Ho, 2010) that is also linked to the presence of bacterial biofilms (Dejea et al., 2014). Altogether, these studies show that biofilms could play a key role in bacterial colonization of the healthy gut and in intestinal diseases.

## Role of mucin components in modulation of bacterial virulence

In addition to acting as a carbon source or as receptors, mucin glycoprotein can influence the expression of different genes implicated in colonization and pathogenicity (Vogt et al., 2015). As example, MUC2 in the mucus layer can play a modulatory role in the pathogenesis of pathogens. Indeed, the ability of *S. enterica* serotype Typhimurium to cause cecal pathology in muc2^−/−^ mice is more dependent on its *invA* gene, coding a *Salmonella* inner membrane protein component of the SPI-1 type 3 secretion system, than it is in wild-type mice (Zarepour et al., 2013). *C. jejuni* can also utilize mucin proteins as a signal to modulate the expression of its virulence factors. Many virulence genes of this pathogen are upregulated in the presence of MUC2 glycoprotein (Tu et al., 2008). Another example is the ability of *V. cholerae* to downregulate the expression of *vps*, coding for its polysaccharide, in response to mucosal signaling and inversely promoting motility in the mucus (Liu et al., 2015). Mucin also activates the two-component sensor histidine kinase ChiS in *V. cholera*. ChiS is the regulator of the chitinases and the chitin utilization pathway, but also plays a role in the virulence of the bacteria since the mutant strain is hypovirulent (Chourashi et al., 2016). Released monosaccharides from mucin O-glycans degradation can also act as a chemical cue to help pathogens to sense their environment and adapt accordingly. As such, sialic acid and GlcNAc are signals that regulate type 1 fimbriae gene expression and curli activity in *E. coli* (Barnhart et al., 2006; Konopka, 2012). GlcNAc and sialic acid also play roles in the virulence of EHEC. In aerobic condition, these mucin-derived sugars inhibit EHEC adhesion to epithelial cells. These amino sugars also repress the expression of genes of the locus of enterocyte effacement (LEE) via the transcriptional regulator NagC involved in the regulation of NAG catabolism (Le Bihan et al., 2017). In contrast, as the sole carbon sources under microaerobic conditions, sialic acid and NAG were shown to stimulate the production of EspB, an effector of the LEE (Carlson-Banning and Sperandio, 2016). EHEC and *C. rodentium* also sense fucose by a two-component system FusKR. It represses the expression of virulence genes while promoting growth (Pacheco et al., 2012; Keeney and Finlay, 2013). Moreover, it was also shown that fucose influences chemotaxis and biofilm formation of *C*. *jejuni* that are important during infection (Dwivedi et al., 2016). Thus, mucus and its derived sugars can play a role in the expression of virulence genes by pathogens.

## Modulation of mucin composition by bacteria

Microbial molecular exchange with the host influences mucin composition. Several bacterial effectors can modulate the expression of mucin by mucus-producing cells (Table 2). Studies using germ-free rats revealed that the presence of microflora through the gastro intestinal tract has a strong and positive influence on the thickness and composition of the mucin (Szentkuti et al., 1990; Enss et al., 1992; Sharma et al., 1995). Different probiotic agents, such as *Lactobacillus* species, can stimulate the production of MUC2 and thereby the secretion of mucin in the intestine, improving pathogen resistance (Mack et al., 1999; Mattar et al., 2002; Caballero-Franco et al., 2007). Other commensal bacteria, such as *B. thetaiotaomicron* can increase the differentiation of goblet cells and their mucus-related gene expression (Wrzosek et al., 2013). Moreover, bacterial fermentation products, such as short-chain fatty acids (SCFAs) like butyrate and propionate enhance the production of MUC2 by the goblet cell in the gut (Barcelo et al., 2000; Burger-van Paassen et al., 2009). This could explain the therapeutic effect of butyrate in colitis where the mucin layer is altered (Finnie et al., 1995). Therefore, commensal residents are important in the maintenance of the mucus layer integrity.

**Table 2:** Effects of bacterial effectors on mucin.

**Bacteria**		**Effector**	**Target**	**Effect on mucin**	**References**
*Campylobacter jejuni*			Distal colonic biopsies	Increased expression of MUC1	Linden et al., 2008
*Clostridium difficile*			Distal colonic biopsies	Increased expression of MUC1	Linden et al., 2008
		ToxA	HT-29 cells	Decrease of mucin exocytosis	Kelly et al., 1994; Branka et al., 1997
*E. coli*	EAEC	Secreted protein Pic	Hog gastric, bovine sub-maxillary and crude mouse large intestine mucin	Mucinase activity / Degradation	Henderson et al., 1999; Harrington et al., 2009
			Goblet cells	Secretagogue activity/Hypersecretion	Navarro-Garcia et al., 2010
	ETEC	Secreted EatA	Purified MUC2	Degradation of MUC2	Kumar et al., 2014
	AIEC (LF82)		T84 cells	Diminished expression of MUC2 and MUC5A	Elatrech et al., 2015
	EHEC (O157:H7)	Adhesion	HT-29 cells	Increased expression of MUC2	Xue et al., 2014
*Lacto-bacillus*	*plantarum* 299v		HT-29 cells	Increased MUC2 secretion	Mack et al., 1999
	*rhamnosus* GG		HT-29 cells	Increased MUC2 secretion	Mack et al., 1999
	*casei* GG		Caco-2 cells	Increased MUC2 secretion	Mattar et al., 2002
*Listeria monocytogenes*		Listeriolysin O (LLO)	HT29-MTX cells	Increased transcription of MUC3, MUC4 and MUC12 Increased secretion of MUC5A	Coconnier et al., 1998; Lievin-Le Moal et al., 2002, 2005
*Salmonella* St Paul			Distal colonic biopsies	Increased expression of MUC1	Linden et al., 2008
*Shigella flexneri*		SST3	Mucin-producing polarized human intestinal epithelial cells	Alteration of glycosylation/ Increased permeability	Sperandio et al., 2013
		Secreted protein Pic	Hog gastric, bovine sub-maxillary, crude mouse large-intestine mucin	Mucinase activity / Degradation	Henderson et al., 1999; Harrington et al., 2009
			Goblet cells	Secretagogue activity / Hypersecretion	Navarro-Garcia et al., 2010
*Vibrio cholerae*		Toxin CT	Goblet cells	Increased mucin secretion	Lencer et al., 1990; Epple et al., 1997
		Secreted TagA	LS174T goblet cell surface mucin	Cleaves mucin glycoproteins	Szabady et al., 2011
*Yersinia enterocolitica*		Virulence Plasmid	Rabbit small intestinal mucin	Degradation/Solubilisation	Mantle and Rombough, 1993

### Modulation of mucin by pathogens

Pathogens have also adapted mechanisms to modulate mucin secretion to enhance pathogenesis by acting on the mucin-secreting cells, altering or inhibiting mucin production (Table 2). One of them is *S. flexneri* that alters the mucus layer through a type III secretion system-dependent manner. This pathogen will act on different elements, such as gene expression, mucin glycosylation and secretion, leading to a less effective mucus barrier (Sperandio et al., 2013). *C. difficile* produces a toxin, ToxA that is responsible for barrier dysfunction and causes severe inflammatory enteritis. ToxA also decreases the mucin exocytosis of colonic mucus-producing cells (Kelly et al., 1994; Branka et al., 1997). The recognition of bacterial components by these cells can also lead to an increased production and secretion of mucin in order to harm the present pathogen. As example, the adhesion of the EHEC O157:H7 to human colon cells HT-29 leads to an increased expression of MUC2 (Xue et al., 2014). Moreover, the cholera toxin of *V. cholerae* and lysteriolysin O of *L. monocytogenes* enhance the secretion of mucin by goblet cells and HT29-MTX cells, respectively (Lencer et al., 1990; Epple et al., 1997; Coconnier et al., 1998; Lievin-Le Moal et al., 2002, 2005). Surprisingly, the Pic protein secreted by *S. flexneri* and enteroaggregative *E. coli* (Henderson et al., 1999; Harrington et al., 2009) is known for its mucolytic activity, but is also a potent mucus secretagogue that induced hypersecretion of mucus by goblet cells (Navarro-Garcia et al., 2010). These studies show how pathogens can affect the behavior of mucus-producing cells in their advantage.

### Mucin degradation by pathogens

Pathogens also developed specific mechanisms to subvert and penetrate the mucus barrier. Some bacteria can directly act on the mucin through a mucinase activity. During enterotoxigenic *E. coli* infections, the autotransporter A (EatA) is involve in mucin degradation and this participate to the delivery of *E. coli* toxins to the cell surface (Kumar et al., 2014). Another example is the adherent and invasive *E. coli* strain LF82, associated with Crohn's disease. LF82 possesses a protease called Vat-AIEC that is implicated in the degradation of mucins and therefore decreases mucus viscosity (Gibold et al., 2016). The Pic autotransporter found in enteroaggregative *E. coli* and *Shigella flexneri* can also degrade various glycoproteins including mucins (Henderson et al., 1999; Harrington et al., 2009). Moreover, the plasmid-bearing *Yersinia enterocolitica*, which contain mucin-degrading enzyme(s), will increase the permeability of the mucus gel layer, allowing the bacteria to move more easily through the mucin (Mantle and Rombough, 1993). *V. cholerae* also produces a secreted protease called TagA that is encoded by the *Vibrio* pathogenicity island (VPI). TagA specifically cleaves mucin glycoproteins and may directly modify host cell surface molecules during *V. cholerae* infection (Szabady et al., 2011). Therefore, to facilitate their infection process, pathogens can directly modify the mucus.

### Inflammation and mucins

Pathogens associated molecular patterns, such as lipopolysaccharide (LPS) and peptidoglycan are also known to stimulate mucin production (Petersson et al., 2011). This stimulation can occur directly on secreting cells, but also be through proinflammatory cytokine production. Recognition of LPS by LPS-binding protein (LBP), CD14, and TLR4 (Toll-Like Receptor) leads to a strong pro-inflammatory response in mammalian cells. LPS has been shown to induce mucin gene expression by binding to TLR4 and LBP (Dohrman et al., 1998; Smirnova et al., 2003). LPS and flagellin from Gram-negative bacteria as well as lipoteichoic acid, a component of the cell wall of Gram-positive bacteria, induce mucin upregulation through the Ras pathway (McNamara and Basbaum, 2001; Theodoropoulos and Carraway, 2007). LPS also increases the production of IL-8 by goblet cells, which leads to secretion of mucin (Smirnova et al., 2003). In addition, pro-inflammatory cytokine IL-6 and TNF-α increase secretion of MUC2, MUC5A, MUC5B, and MUC6 b*y* the intestinal cell line *LS180* despite a reduced glycosylation (Enss et al., 2000). Inflammation could be one of the aspects affecting the integrity of the mucus layer in inflammatory bowel diseases. Furthermore, the AIEC strain LF82 is able to alter the expression of the mucin gene and IL-8 of colonic cells T84 that could also lead to a defective mucus layer (Elatrech et al., 2015). Thus, pathogens can also alter the mucus production indirectly, through inflammation.

## Conclusion

Intestinal bacteria have adapted to colonize the mucus layer by adhering to intestinal mucus components, using mucus-derived nutrients and sensing chemical cues for adaptation. In many ways, pathogenic bacteria have used these strategies for successful infection. There has been growing recognition of the important role played by the mucus barrier and microbiota and their interaction with the pathogens in regulating the severity of infectious diseases. But, the precise mechanisms by which enteric bacterial pathogens interact with mucus components in combination with the microbiota activity are being investigated. As the mucus layer acts as a first line of defense against enteric bacteria, further investigations are needed to understand the interactions between pathogens, microbiota and the mucus layer, in order to develop efficient therapeutic strategies. Identifying and characterizing specific mucin signal(s) and corresponding regulatory adaptation and virulence responses could contribute to the development of new anti-infective strategies. In doing so, other weapons could be added to the arsenal against intestinal pathogens.

## Author contributions

All authors listed have made a substantial, direct and intellectual contribution to the work, and approved it for publication. The manuscript was written by J-FS and JH and was duly revised by GLB, PV and MJ.

### Conflict of interest statement

The authors declare that the research was conducted in the absence of any commercial or financial relationships that could be construed as a potential conflict of interest.

## Abbreviations

MLG: Mucus gel layer
A/E: Attaching and effacing
NAG: *N*-acetyl-D-glucosamine
NANA: *N*-acetylneuraminic acid
EHEC: Enterohemorrhagic *E. coli*
MUB: Mucus-binding proteins
LAB: Lactic acid bacteria
HBGA: Histoblood group antigen
SIgA: Secretory IgA
AIEC: Adherent invasive *E. coli*
LPB: LPS-binding protein
TLR: Toll-like receptor
VPI: *Vibrio* pathogenicity island.
